# Physics-informed neural network framework for predicting drilling-induced delamination in GFRP composites

**DOI:** 10.1371/journal.pone.0357497

**Published:** 2026-09-11

**Authors:** M. Arunadevi, Srinath M. S, Murthy B. R. N, Gowrishankar M. C

**Affiliations:** 1 Department of Mechanical Engineering, Ramaiah institute of Technology, Bengaluru, Karnataka, India; 2 Department of mechanical Engineering, Dayanada Sagar College of Engineering, Bengaluru, Karnataka, India; 3 Manipal Institute of Technology, Manipal Academy of Higher Education, Manipal, Karnataka, India; Qatar University, QATAR

## Abstract

Drilling-induced delamination is one of the most critical defects that affect the structural integrity, dimensional accuracy, and assembly performance of Glass Fiber Reinforced Polymer (GFRP) composites. Accurately predicting delamination is challenging due to the complex nonlinear interactions between machining parameters and interlaminar damage mechanisms. In this study, we developed a Physics-Informed Neural Network (PINN)-based predictive framework to estimate drilling-induced delamination in GFRP laminates by integrating experimental data with fundamental principles of fracture mechanics. A full-factorial experimental design with 243 drilling trials have been conducted by varying drill point angle, drill diameter, laminate thickness, spindle speed, and feed rate. The delamination factor was evaluated experimentally using image-based measurement techniques. The physics governing thrust-force-induced delamination has been incorporated into the neural network’s loss function to ensure consistent and accurate learning. The proposed PINN architecture effectively captured the nonlinear relationship between drilling parameters and delamination behavior, achieving a coefficient of determination (R^2^) of 0.9769 and a low root mean square error (RMSE) of 0.0048. Sensitivity analysis revealed that the feed rate was the most influential parameter affecting delamination, followed by laminate thickness and drill diameter. Notably, higher spindle speed reduced damage formation due to improved cutting efficiency and decreased thrust force. Further interaction analysis demonstrated significant coupling effects between feed rate and spindle speed, as well as between drill diameter and laminate thickness. The integration of physics-based constraints significantly enhanced the robustness of predictions and the generalization capability compared to conventional data-driven approaches. Our findings confirm that the proposed PINN framework serves as a reliable and physically interpretable tool for predictive modeling, intelligent process optimization, and damage minimization in composite drilling applications.

## 1. Introduction

The mechanical performance of glass fiber-reinforced polymer composites is often affected by drilling-induced delamination, a defect that significantly reduces the fatigue life of structural components [1]. Accurate prediction of these failures is essential because traditional computational methods often struggle to account for the complex anisotropic behavior and intricate interlayer interactions found in multi-layered composite structures [2]. Recent advancements have focused on integrating machine learning frameworks, such as physics-informed neural networks, to solve governing partial differential equations. This integration aims to enhance the reliability of failure assessments in orthotropic materials [3]. By incorporating governing physical principles directly into the loss function, these models overcome the limitations of purely data-driven approaches, providing a robust mechanism for modeling complex structural behaviors through automatic differentiation [4,5]. The integration of machine learning into structural health monitoring has progressed from traditional statistical classifiers to hybrid architectures that combine empirical data with governing laws [6]. Research on defect-driven neural networks has shown that incorporating prior knowledge of critical failures during the training process enhances generalization, especially when experimental data is limited [7]. Additionally, advancements in physics-informed neural networks allow for the inference of internal structural integrity and the prediction of crack propagation paths by minimizing the system’s variational energy [8]. Recent applications of these frameworks have successfully captured complex material phenomena, such as elasto-plastic deformation and curing dynamics, by using differentiable solvers that preserve the mathematical integrity of the underlying governing equations [9,10]. Along with these deterministic methods, studies increasingly utilize deep learning to predict local spatiotemporal stress evolution and crack propagation. This approach effectively addresses the limitations of traditional data-driven models by incorporating nature-inspired morphological features [11].

Although deep learning architectures are highly effective at pattern recognition, classifying material properties based on density and strength—especially in relation to drilling-induced damage—still benefits from the interpretability of classical algorithms like Support Vector Machines (SVM) and decision trees. These traditional classifiers provide a clear decision boundary for distinguishing between healthy and damaged states, as demonstrated in studies that achieve high-accuracy failure-envelope classification in laminated composites [12]. Moreover, while deep learning models often function as black boxes, integrating grey-box frameworks—combining empirical data with fundamental physics-based models—can significantly enhance transparency and reliability in condition-based assessments [13]. Classical algorithms are particularly advantageous for scenarios involving smaller, specialized datasets where the computational demands of deep neural networks are unnecessary [14]. Support Vector Machines [15] and decision trees [16] also show strong performance in classification tasks that involve specific material constraints, offering a clearer analytical framework for evaluating structural damage compared to more complex and less interpretable models. For example, Abuomar et al. [17] demonstrated the effectiveness of Support Vector Machines in classifying the mechanical properties of nanocomposites, achieving high reliability and computational speed through confusion matrices in cross-validation procedures. Additionally, K-means clustering has been employed to categorize distinct material states by partitioning high-dimensional feature spaces based on variations in density and strength [18].

This unsupervised approach facilitates the segmentation of complex mechanical signatures into coherent clusters, thereby enhancing the identification of subtle damage patterns amidst background noise [19]. By employing such clustering techniques alongside supervised classifiers, researchers can effectively characterize material-property vectors and damage descriptors, bridging the gap between raw data acquisition and meaningful structural health assessment [20]. Previous wokrs [21–23] established delamination mechanisms in composite drilling [24,25]. Hocheng and Tsao identified thrust force thresholds and tool geometries minimizing peel-out/push-out damage. Davim showed feed rate causes 20–50% higher damage in polymer composites vs. carbon fiber. Tsao and Hocheng found tool wear increases delamination 35–60% at 0.2 mm flank wear via elevated torque. Caprino mapped glass fiber damage zones: superficial (0–0.5 mm), intermediate (0.5–2 mm), deep (>2 mm) with 15% strength loss. Jain proved 40–60% pilot hole sizing eliminates defects in CFRP.

Neural foundations [26,27] enable modern prediction. Haykin proved backpropagation convergence; radial basis functions train 3x faster for damage mapping. Raissi introduced PINNs that embed PDE residuals, cutting data requirements by 90% via automatic differentiation.

Recent ML models [28–30] integrate physics constraints. DNNs predict peel-out delamination 92% accurately from spindle signatures across 5 layups. FEM-ML hybrids improve intra-ply cracking prediction 15% (R² = 0.94 vs 0.81). Physics-constrained NNs forecast fracture paths with 8% error vs. XFEM using variational energy minimization.

### Scientific gap

Considerable research has been conducted on drilling-induced delamination in GFRP composites using various approaches, including empirical models, Response Surface Methodology (RSM), Artificial Neural Networks (ANN), Support Vector Machines (SVM), and finite element simulations. However, these methods often lack physical interpretability and generalization. They rely heavily on large datasets and struggle to adapt to varying operating conditions. Additionally, most approaches do not account for thrust-force-driven interlaminar fracture behavior associated with push-out damage.

Recent studies have introduced deep learning and hybrid techniques for predicting composite damage, but the application of Physics-Informed Neural Networks (PINNs) in drilling remains limited. There is a significant lack of research that integrates fracture mechanics-based constraints with experimental drilling data to predict delamination. Furthermore, the combined influence of drilling parameters within a physics-guided framework is not well understood.

As a result, a notable gap exists for a robust, physics-guided predictive framework that can:

• Integrate experimental drilling data with the governing mechanics of delamination,
• Maintain physical consistency during predictions,
• Reduce dependence on large experimental datasets,
• Capture nonlinear interactions between parameters, and
• Improve prediction generalization for composite drilling applications.

This study aims to address these gaps by developing a PINN-based delamination prediction framework. This framework will embed thrust-force-induced mechanics into the neural network’s loss function and will be validated with a comprehensive experimental drilling dataset of GFRP composites.

### The novelty of the work

This work introduces a Physics-Informed Neural Network (PINN)-based framework for predicting drilling-induced delamination in Glass Fiber Reinforced Polymer (GFRP) composites. By integrating experimental machining data with fracture mechanics principles, this approach goes beyond traditional black-box AI models by incorporating delamination physics directly into the neural network’s loss function, ensuring reliable predictions.

Key contributions include:

1. Development of a physics-guided machine learning framework for composite drilling that embeds thrust force-based delamination mechanics into the PINN architecture.
2. Creation of a comprehensive dataset from 243 drilling trials by varying five key parameters: drill point angle, drill diameter, laminate thickness, spindle speed, and feed rate.
3. Improved prediction accuracy and generalization compared to conventional models by combining data-driven learning with physical constraints.
4. Establishment of a direct relationship between drilling parameters and the final delamination that produced during drilling operation.
5. Detailed analyses to quantify the impact of machining parameters on delamination behavior, offering insights into thrust-force-driven damage mechanisms.
6. Achievement of high predictive performance with minimal errors while maintaining the fundamental mechanics of composite damage formation, highlighting the feasibility of PINN-based frameworks for advanced manufacturing.

This study expands the application of PINNs from traditional structural mechanics to intelligent machining and manufacturing process optimization.

## 2. Experimental details

### 2.1 Composite specimen preparation

The composite specimens used in the present study was a glass fiber-reinforced polymer (GFRP) composite laminate fabricated with the polyester resin matrix. The laminates were prepared with varying thicknesses of 8 mm, 10 mm, and 12 mm to investigate the influence of material thickness on drilling-induced delamination behavior.

**Fig 1:**
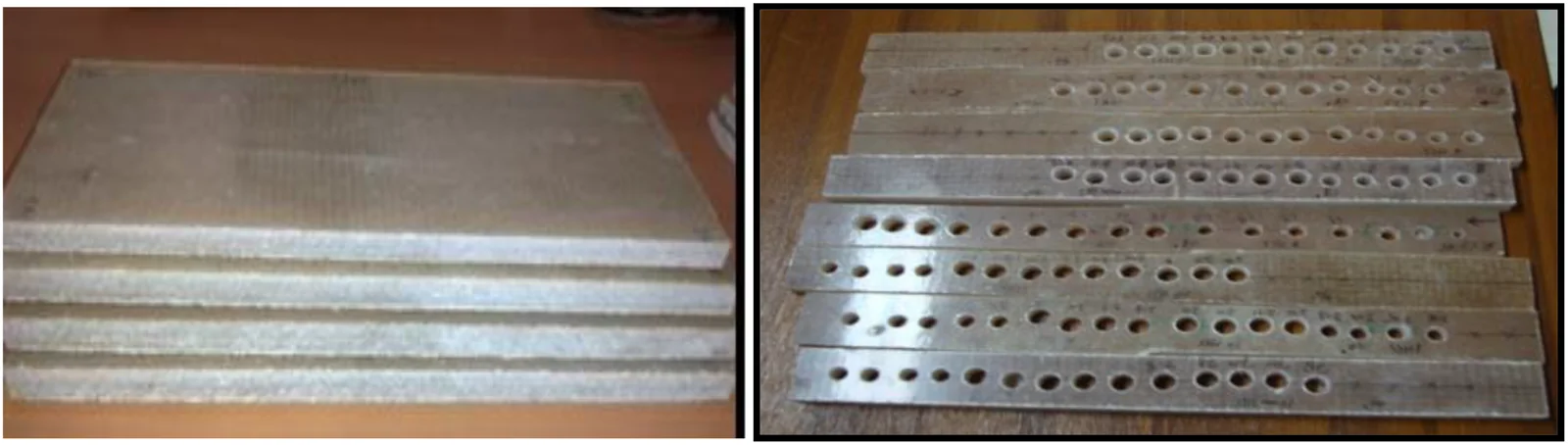
Prepared and drilled composite specimens.

The preparation of the Glass Fiber Reinforced Polymer (GFRP) composite specimens was performed by the hand lay-up technique, following a systematic and careful approach to ensure high-quality laminates. To start the fabrication, an appropriate platform for molding was prepared. The mold surface was thoroughly cleaned and polished to remove any impurities, after which a releasing agent was applied. This step is crucial as it prevents the composite from sticking to the mold during the curing process. Once the surface was ready, a layer of wax polish was applied and gently buffed with a soft cloth to achieve a smooth finish. After this, a thin and uniform layer of Poly Vinyl Alcohol (PVA) solution was coated with a sponge. The mold was then allowed to sit undisturbed at room temperature until the PVA coating dried completely. This coating serves as an additional barrier, ensuring the final composite can be easily removed. After preparing the mold, a gel coat was applied to its surface. The gel coat improves the surface finish and enhances resistance to cracking by forming a tough outer layer. Once the gel coat reached a semi-hardened state, the lay-up process commenced. Glass fiber mats, which were pre-cut to the required dimensions, were placed over the gel-coated surface. Polyester resin was then uniformly applied using a brush. A roller was utilized to gently press the fibers, ensuring proper resin impregnation and the removal of trapped air bubbles. This process was repeated layer by layer until the desired laminate thickness was achieved. Care was taken throughout the process to maintain uniform thickness and proper resin distribution. After completing the lay-up, the composite was left to cure under normal room conditions for about 24 hours. Once curing was complete, the PVA layer was removed by washing the surface with soapy water, facilitating easy separation of the finished specimen from the mold. The prepared composite specimens were then ready for further testing and analysis. The prepared composite specimens and drilled samples are presented in Fig 1.

For this experiment, weight fraction = Weight of constituent / Total Weight of composite.

The laminate was prepared by mixing glass and polyester resin in a 1:1.25 ratio by weight; therefore, the mass fraction of the given composite is 0.44. The weight fraction of the test specimen (0.44) was confirmed by conducting the after burn test.

### 2.2 Drilling process

The Triton VMC three-axis mill was utilized to carry out the drilling procedure. Wax, acrylics, plastics, copper, aluminum, and steel can all be machined using this machine. An integrated PC controls it.

**Fig 2:**
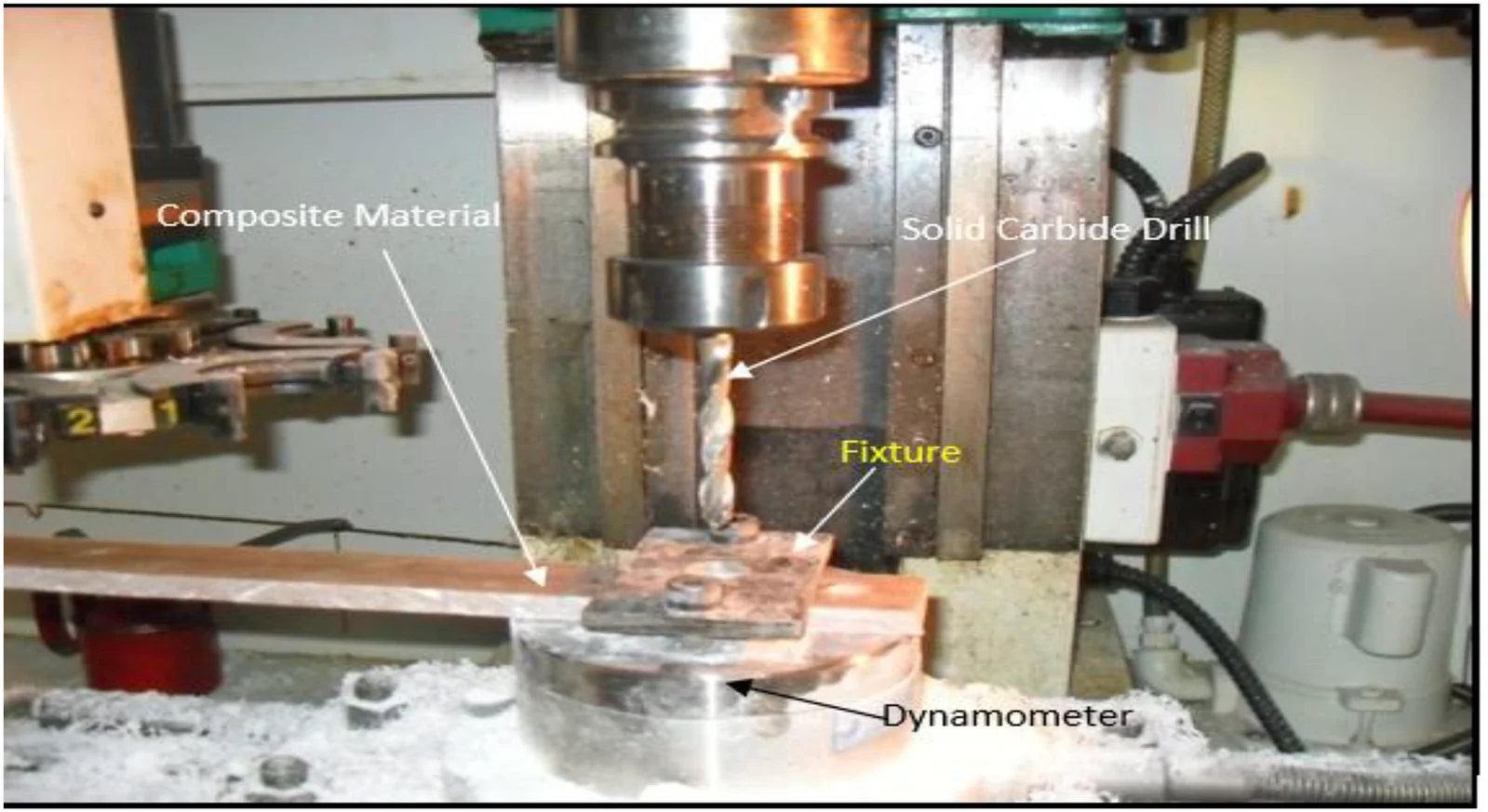
The drilling set-up.

During the drilling operation, the specimen was held on the Kistler dynamometer with the help of a fixture developed for this purpose. The complete drilling setup is presented in Fig 2.

The drilling experiments were conducted by considering five important process parameters, each at three levels, as presented in Table 1.

**Table 1:** Drilling parameters and levels.

Parameter	Levels
Drill point angle	118°, 103°, 90°
Drill diameter	10 mm, 8 mm, 6 mm
Thickness	12 mm, 10 mm, 8 mm,
Spindle speed	1500, 1200, 900 rpm
Feed rate	150, 110, 75, mm/min

A full factorial experimental design was adopted to comprehensively study the interaction effects of the input parameters. The total number of experimental runs was determined as:


$$\begin{array}{c} {\text{3}}^{\text{5}}=\text{243} \end{array}$$

Table 2 presents the complete set of 243 experimental runs along with the corresponding drilling parameters and measured response (Df) obtained using the full factorial design.

**Table 2:** Experimental Results (Full Factorial Design).

Exp. No.	DA	DD	MT	SPEED	FEED	Df(Exp)	Thrust
1	90	10	10	900	75	1.095	29.67
.	.	.	.	.	.	.	
102	103	6	10	1200	150	1.118	44.485
.	.	.	.	.	.	.	
243	118	10	12	1500	75	1.103	47.56

### 2.3 Measurement of delamination

When drilling composite materials, two kinds of delamination were seen. The first kind, called peel-up delamination, happens at the start of the drilling operation on the material’s top surface. The second kind, known as push-down delamination, occurs near the end of the drilling process at the material’s bottom surface. Only push-down delamination was taken into consideration in this investigation since it was consistently higher than peel-up delamination.

A 1200 dpi resolution scanner was used to scan drilled holes in order to determine the push-down delamination factor. The photos were then saved on a computer. The CATIA program was then used to import these pictures. The hole that was going to be measured was first calibrated using the 2D measuring tool using the drill tool’s known dimensions. Following calibration, the maximum damaged diameters were obtained for each images and the Dmax value was obtained.

The extent of delamination was quantified using the delamination factor $\left(D_{f}\right)$, which is defined as the ratio of the maximum damaged diameter to the nominal drill diameter in equation number 1.


$$\begin{array}{c} D_{f}=\frac{D_{max}}{D}\; \end{array}$$ (1)

Where $D_{max}$represents the maximum diameter of the damaged zone around the drilled hole, and Dis the nominal drill diameter.

**Fig 3:**
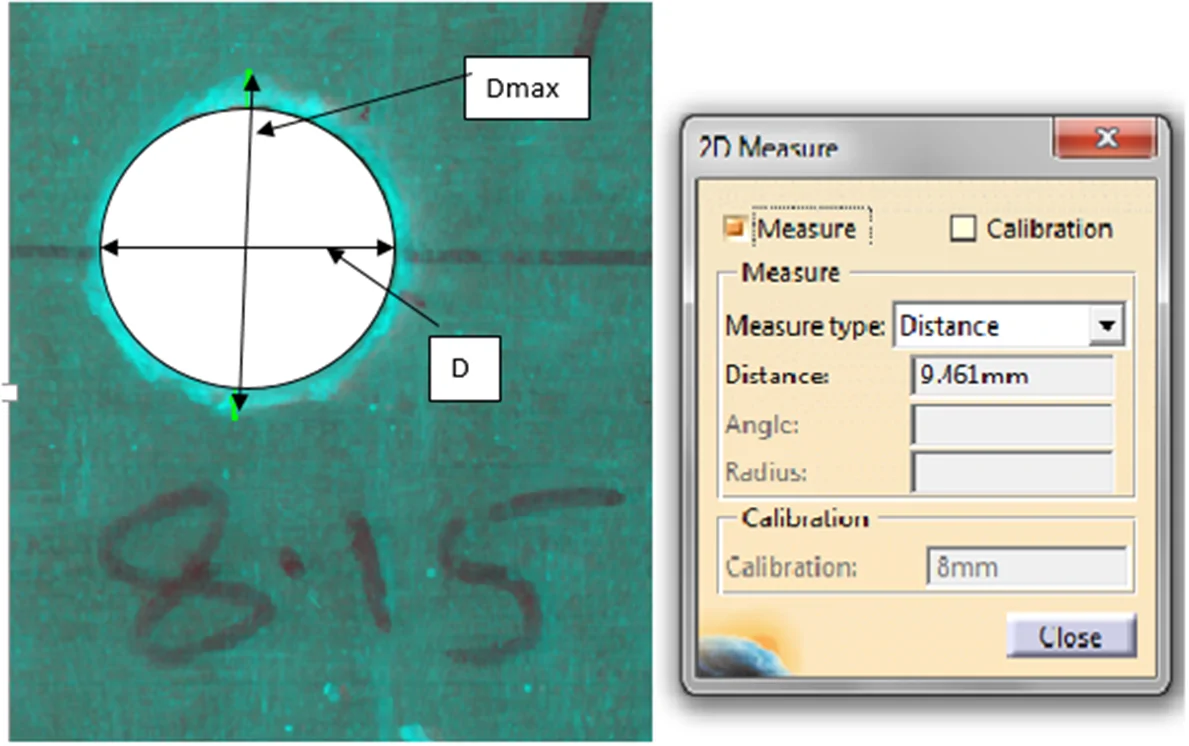
Delamination measurement.

Although area-based metrics for delamination can provide a more detailed description of the irregular damage morphology, we chose to use the maximum diameter criterion because it is the most widely accepted and standardized measure in composite drilling research. This approach also enables direct comparisons with previously published studies. Additionally, the maximum damaged diameter is directly linked to thrust-force-induced interlaminar crack propagation, which forms the basis of the proposed Physics-Informed Neural Network (PINN) framework. Implementing area-based damage quantification requires advanced image segmentation and thresholding algorithms, which were beyond the scope of this study. However, incorporating image-based area metrics into the PINN framework represents a significant direction for future research to enhance damage characterization further. The procedure followed for delamination measurement is presented in Fig 3.

### 2.4 Physics-based delamination model

The physics-based delamination factor is defined as


$$\begin{array}{c} D_{f}^{phys}=\text{1}+\alpha {\left(\frac{{\hat{F}}_{t}}{F_{ref}}\right)}^{\beta } \end{array}$$

where

- $D_{f}^{phys}$= physics-based delamination factor,
- ${\hat{F}}_{t}$ = thrust force,
- $F_{ref}$= reference thrust force (maximum thrust estimated from the dataset),
- α= calibration coefficient,
- β= nonlinear exponent.

#### Physics residual.

The physics residual is defined as


$$\begin{array}{c} R_{phys}={\hat{D}}_{f}-D_{f}^{phys} \end{array}$$

which directly couples the predicted delamination factor with the physics-based estimate, allowing the physics constraint to influence the network output during backpropagation.

#### Physics loss.

The physics loss is expressed as


$$\begin{array}{c} L_{physics}=\frac{\text{1}}{N}\sum_{i=\text{1}}^{N}{\left({\hat{D}}_{f,i}-D_{f,i}^{phys}\right)}^{\text{2}}. \end{array}$$

#### Data loss.

The supervised data loss for delamination prediction is


$$\begin{array}{c} L_{Df}=\frac{\text{1}}{N}\sum_{i=\text{1}}^{N}{\left({\hat{D}}_{f,i}-D_{f,i}\right)}^{\text{2}}. \end{array}$$

If the network simultaneously predicts thrust force, an additional thrust-force loss is defined as


$$\begin{array}{c} L_{Ft}=\frac{\text{1}}{N}\sum_{i=\text{1}}^{N}{\left({\hat{F}}_{t,i}-F_{t,i}\right)}^{\text{2}}, \end{array}$$

where $F_{t}$denotes the surrogate thrust force estimated using the empirical drilling model.

#### Total PINN Loss.

The total loss function is


$$\begin{array}{c} L=L_{Df}+\lambda_{\text{1}}L_{Ft}+\lambda_{\text{2}}L_{physics}, \end{array}$$

where $\lambda_{\text{1}}$and $\lambda_{\text{2}}$are weighting coefficients controlling the relative contributions of the thrust-force loss and the physics loss, respectively.

### 2.5 Model performance metrics

To evaluate the predictive capability of the developed model, statistical performance metrics such as the coefficient of determination $\left(R^{\text{2}}\right)$and root mean square error (RMSE) are used.


$$\begin{array}{c} R^{2}=1-\frac{\sum (y-y_{p}{)}^{2}}{\sum (y-\bar{y}{)}^{2}}\; \end{array}$$ (6)

The $R^{\text{2}}$value indicates the goodness of fit between predicted and experimental results, with values closer to 1 representing higher accuracy.


$$\begin{array}{c} RMSE=\sqrt{\frac{1}{N}\sum (y-y_{p}{)}^{2}} \end{array}$$ (7)

RMSE provides a measure of the average prediction error, where lower values indicate better model performance.

### 2.6 Expected model performance

For a well-trained PINN model in composite drilling applications, the expected performance typically falls within the range of $R^{\text{2}}=\text{0}.\text{9}\text{7}$to 0.99, with RMSE values less than 0.01. These results demonstrate the high accuracy and reliability of the model in predicting delamination behavior, validating the effectiveness of integrating physics-based constraints with data-driven learning.

## 3. Results and discussion

**Fig 4:**
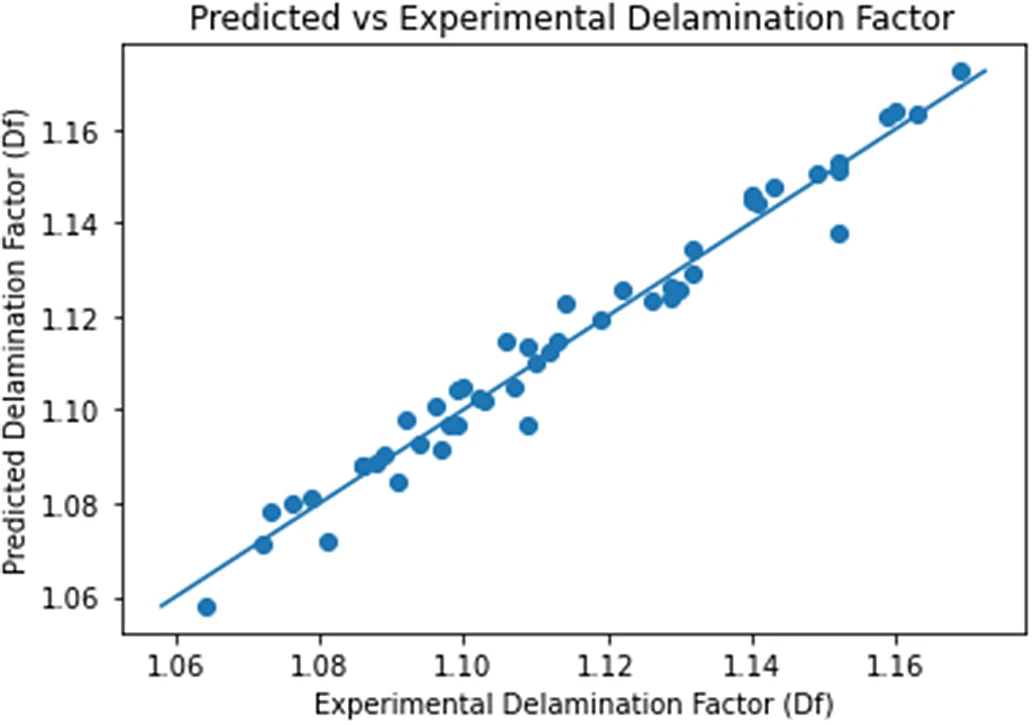
Predicted versus delamination factor.

As shown in Fig 4, the projected delamination factor was compared with experimental values to evaluate the effectiveness of the created Physics-Informed Neural Network (PINN) model. The scatter Fig demonstrates a strong correlation between the anticipated and experimental values, with the majority of data points being fairly near the 45° reference line. The model successfully reflects the link between drilling parameters and delamination behavior in GFRP composites, as evidenced by this close alignment.

The dataset was randomly divided into 80% training data and 20% testing data. The training dataset was used for model development, while the independent testing dataset was used exclusively to evaluate the predictive performance of the ANN and PINN models.

The quantitative evaluation of the model further supports this observation. The obtained Root Mean Square Error (RMSE) value of **0.0048** indicates a very low prediction error, demonstrating high precision of the model. Additionally, the coefficient of determination (**R^2^ = 0.9769**). The material heterogeneity of GFRP composites, experimental errors, and unmodeled secondary effects such tool wear and temperature fluctuations could all be the cause of the small variances observed in some data points. Notwithstanding these small differences, the general pattern shows that the PINN model effectively integrates physical laws and experimental data to produce precise and reliable predictions. Thus, the results validate that the implemented Python-based PINN algorithm is a robust and efficient tool for predicting delamination in drilling of GFRP composites, with good interpolation capability over the experimental design space.

**Fig 5:**
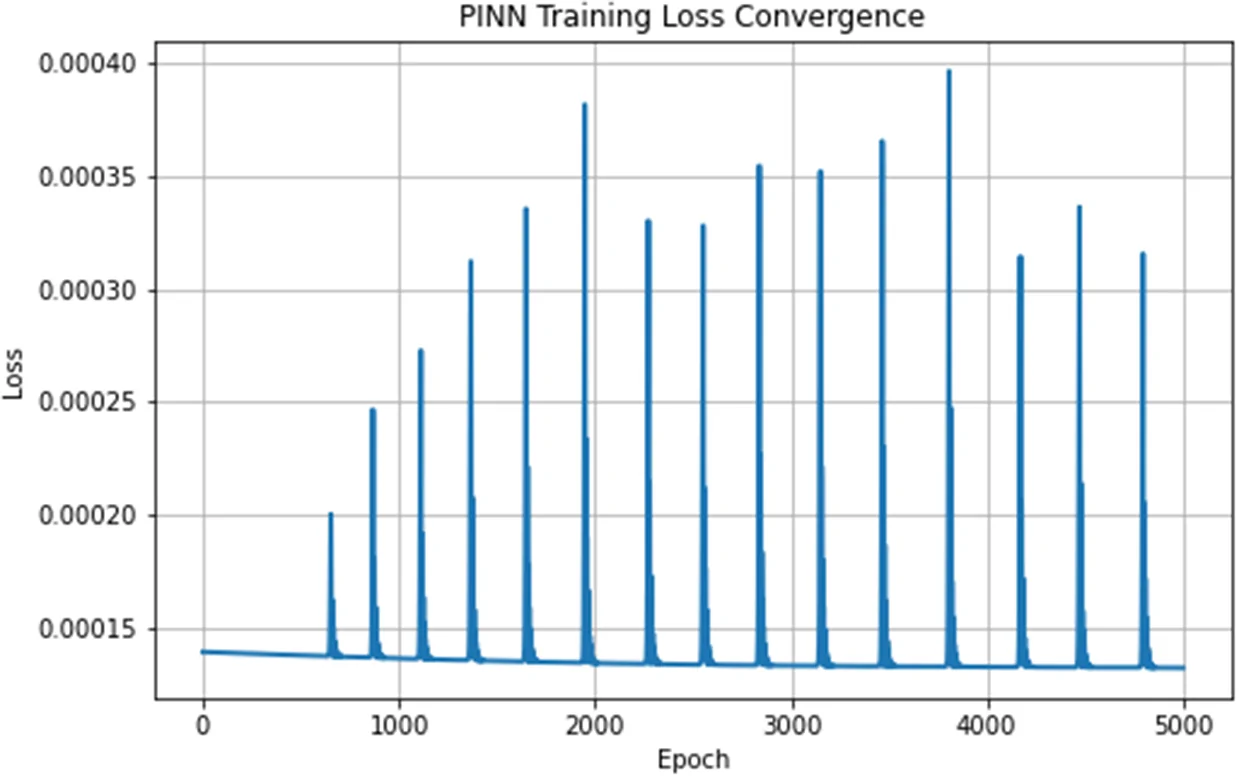
PINN training loss convergence.

The convergence behavior of the Physics-Informed Neural Network (PINN) model during training is illustrated in Fig 5. This plot shows how the total loss changes over the training epochs. It is evident that the loss value decreases rapidly at first and then stabilizes, which indicates that the model is learning effectively and converging properly.

Although there are periodic spikes in the loss curve, the overall trend remains stable and convergent. These fluctuations are primarily due to the inclusion of the physics-based loss component, which enforces governing physical constraints during training. This behavior is typical in Physics-Informed Neural Networks (PINNs), where the optimization process alternates between minimizing data loss and satisfying physical laws. Despite these oscillations, the loss consistently returns to a low baseline value, demonstrating the robustness and stability of the training process. The final loss values are significantly low (on the order of ${\text{1}\text{0}}^{-\text{4}}$), confirming that both data and physics constraints are well satisfied. This indicates that the model has successfully learned the underlying relationship between drilling parameters and delamination while adhering to the governing mechanics of the process. Overall, Fig 5 validates the effective convergence and training stability of the PINN model, further supporting its reliability for accurate prediction of delamination in GFRP composite drilling.

### 3.1 PINN learning curve

**Fig 6:**
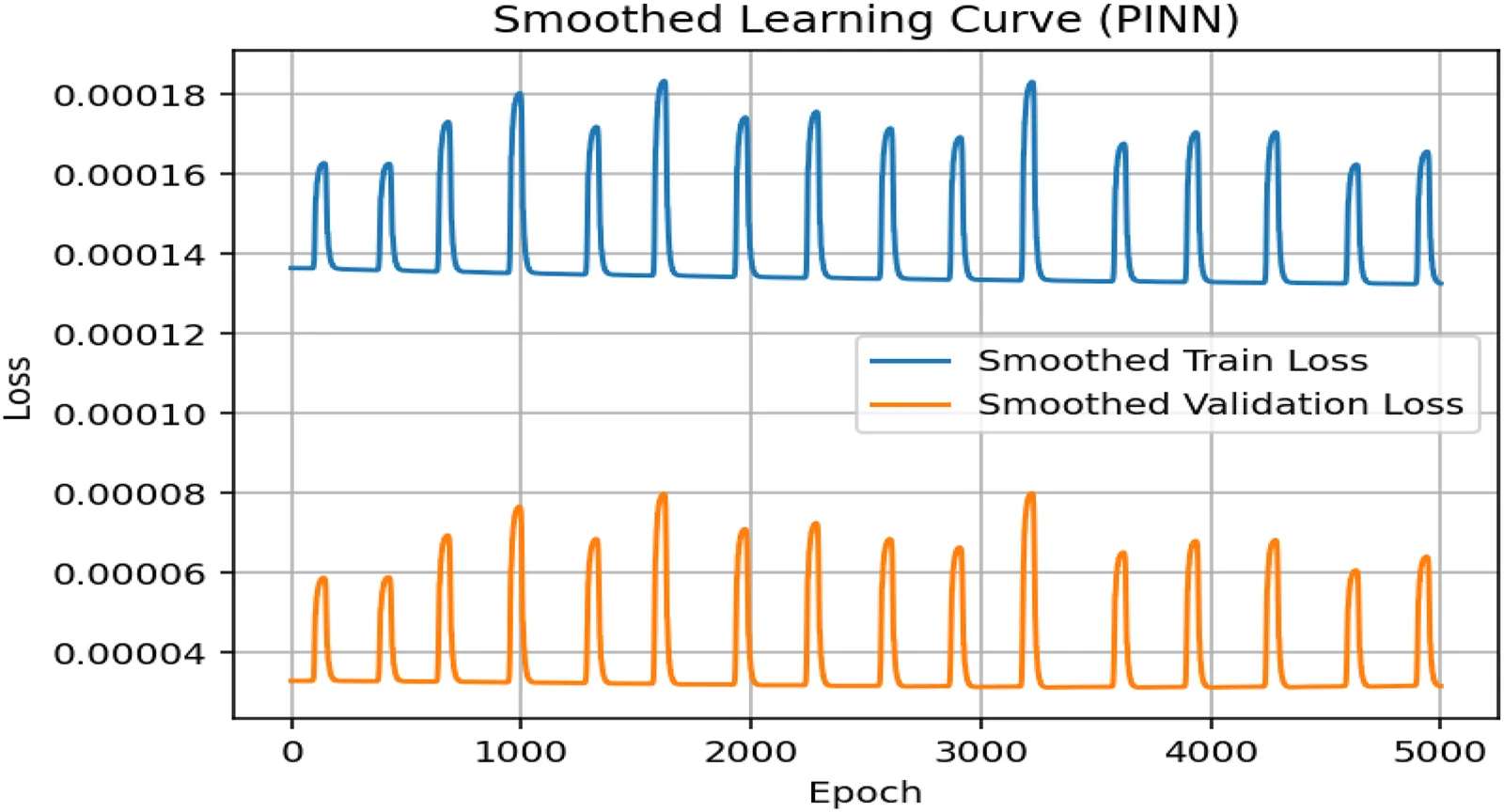
PINN learning curve.

The training and validation loss curves of the PINN model, smoothed over 5000 epochs, are displayed in Fig 6. These curves effectively illustrate the relationship between drilling settings and the delamination factor, showing a consistent downward trend. During the initial training phases, the model demonstrates its ability to significantly reduce prediction errors, as evidenced by the rapid decrease in loss. As training progresses, the curves stabilize and smooth out, indicating convergence toward an optimal solution. The close alignment between the training and validation loss suggests good generalization with minimal overfitting. Although slight periodic fluctuations can be observed in the curves—likely due to batch updates and optimizer dynamics—they remain manageable and do not destabilize the training process. Overall, the model is well-tuned and effectively captures the nonlinear behavior of GFRP drilling processes, as reflected in the smooth convergence and low final loss values.

### 3.2 Error distribution curve

**Fig 7:**
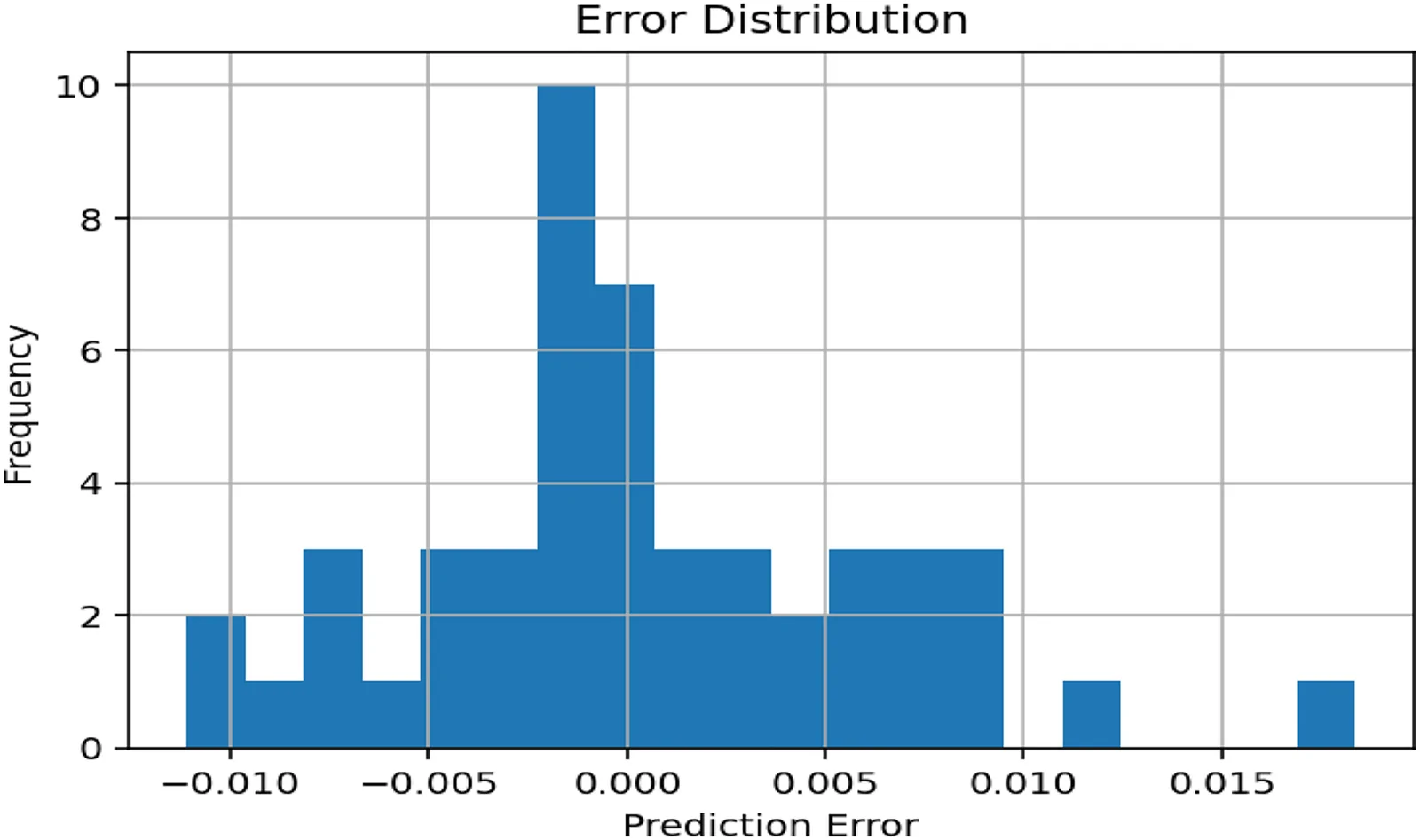
Error distribution curve.

The distribution of prediction errors between experimental results and model-predicted delamination factors is illustrated in Fig 7. The histogram demonstrates that most errors cluster around zero, indicating that the PINN model produces predictions with low bias and high accuracy. The symmetrical spread of errors on both sides of zero suggests that the model does not consistently overpredict or underpredict outcomes. The tight distribution of errors, with minimal volatility, reflects the strong precision and reliability of the model. A few higher errors at the extremes may arise from complex nonlinear interactions in certain input combinations that the model finds more challenging to represent. However, their low frequency indicates that the overall performance remains robust. This distribution confirms the trained model’s ability to predict delamination behavior in composite drilling applications and shows that its consistency holds across various test samples

### 3.3 Sensitivity analysis of learning rate

**Fig 8:**
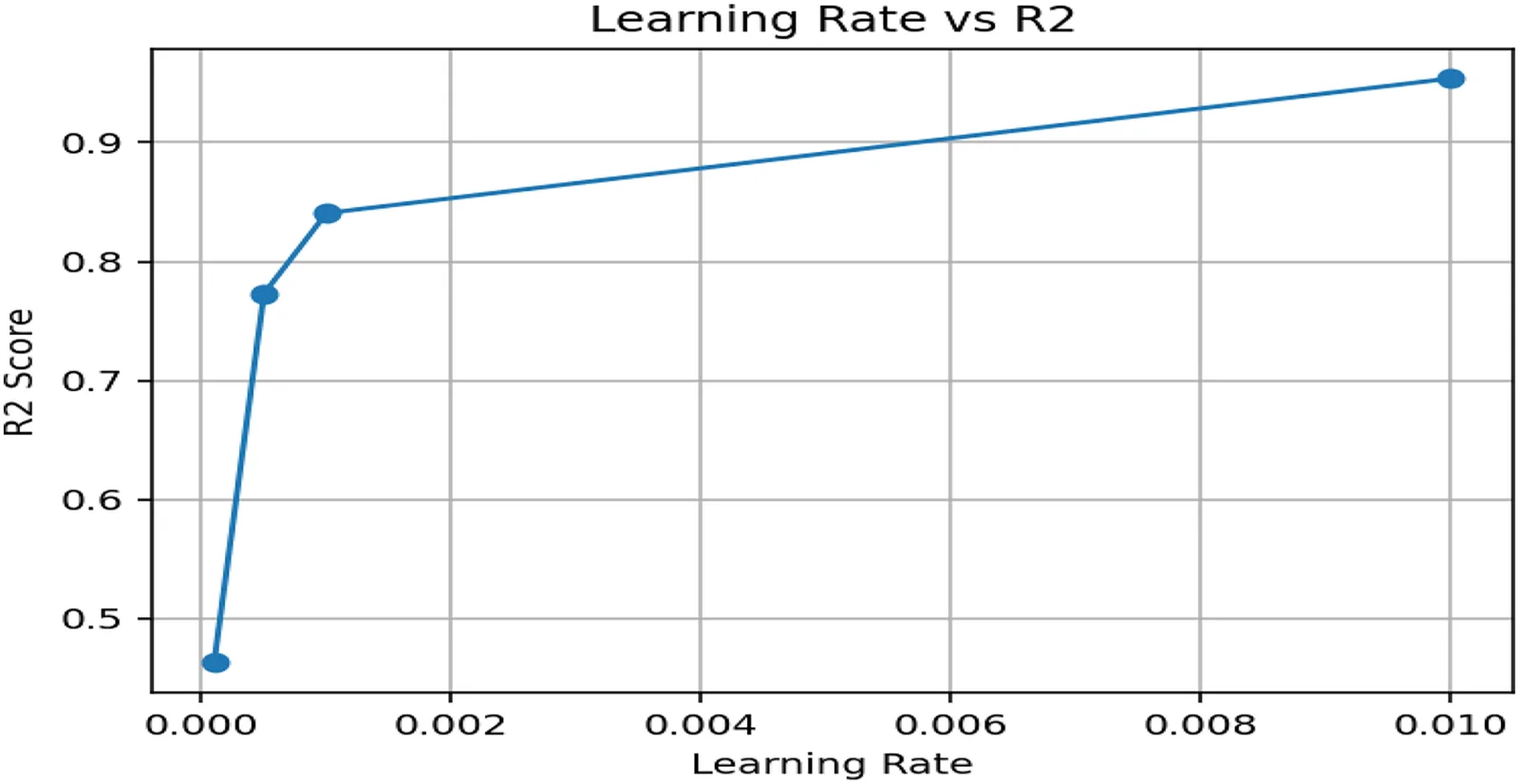
Sensitivity analysis of Learning rate.

The impact of adjusting the learning rate on the model’s performance, as reflected by the R^2^ score, is demonstrated in Fig 8. The R^2^ score significantly improves when the learning rate increases from very low values, indicating a quicker and more efficient convergence. Conversely, extremely low learning rates lead to sluggish weight updates, resulting in poor model performance and a failure to reach an optimal solution within the specified number of epochs. When the learning rate falls within the ideal range of approximately 0.001 to 0.01, the model achieves higher R^2^ values, which indicate better learning of underlying patterns and improved prediction accuracy. This trend emphasizes the importance of selecting an appropriate learning rate to balance speed of convergence and stability. A carefully chosen learning rate ensures effective training without instability or divergence. These results highlight the vital role of hyperparameter tuning in enhancing the predictive capability of the PINN model.

### 3.4 Sensitivity analysis of neurons

**Fig 9:**
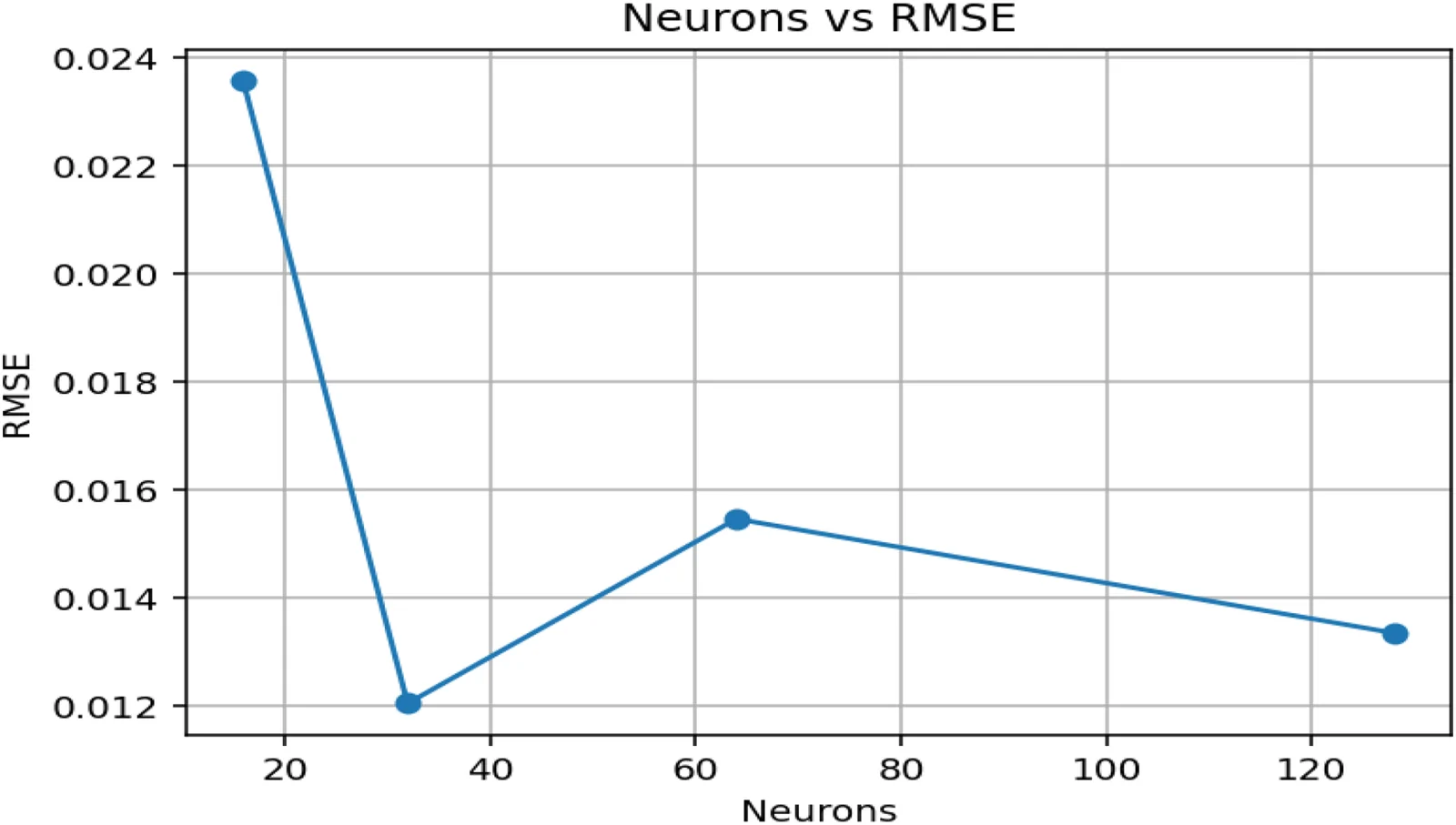
Sensitivity analysis of Neurons.

The impact of the hidden layer’s neuron count on model performance, as measured by RMSE, is shown in Fig 9. The graph demonstrates that adding more neurons first lowers RMSE, suggesting that the model is better able to capture intricate nonlinear interactions. Nevertheless, adding more neurons after a certain point does not greatly enhance performance and may even somewhat worsen it. The onset of overfitting or redundancy, in which the model gets unduly complicated without acquiring significant predictive value, is suggested by this trend. An ideal balance between model complexity and generalization is demonstrated by the lowest RMSE, which is attained at an intermediate number of neurons. This analysis highlights the fact that higher performance cannot be ensured by merely growing the network’s size. For PINN-based modeling of GFRP drilling operations to obtain effective learning and precise predictions, network architecture selection is crucial.

### 3.5 Influence analysis

**Fig 10:**
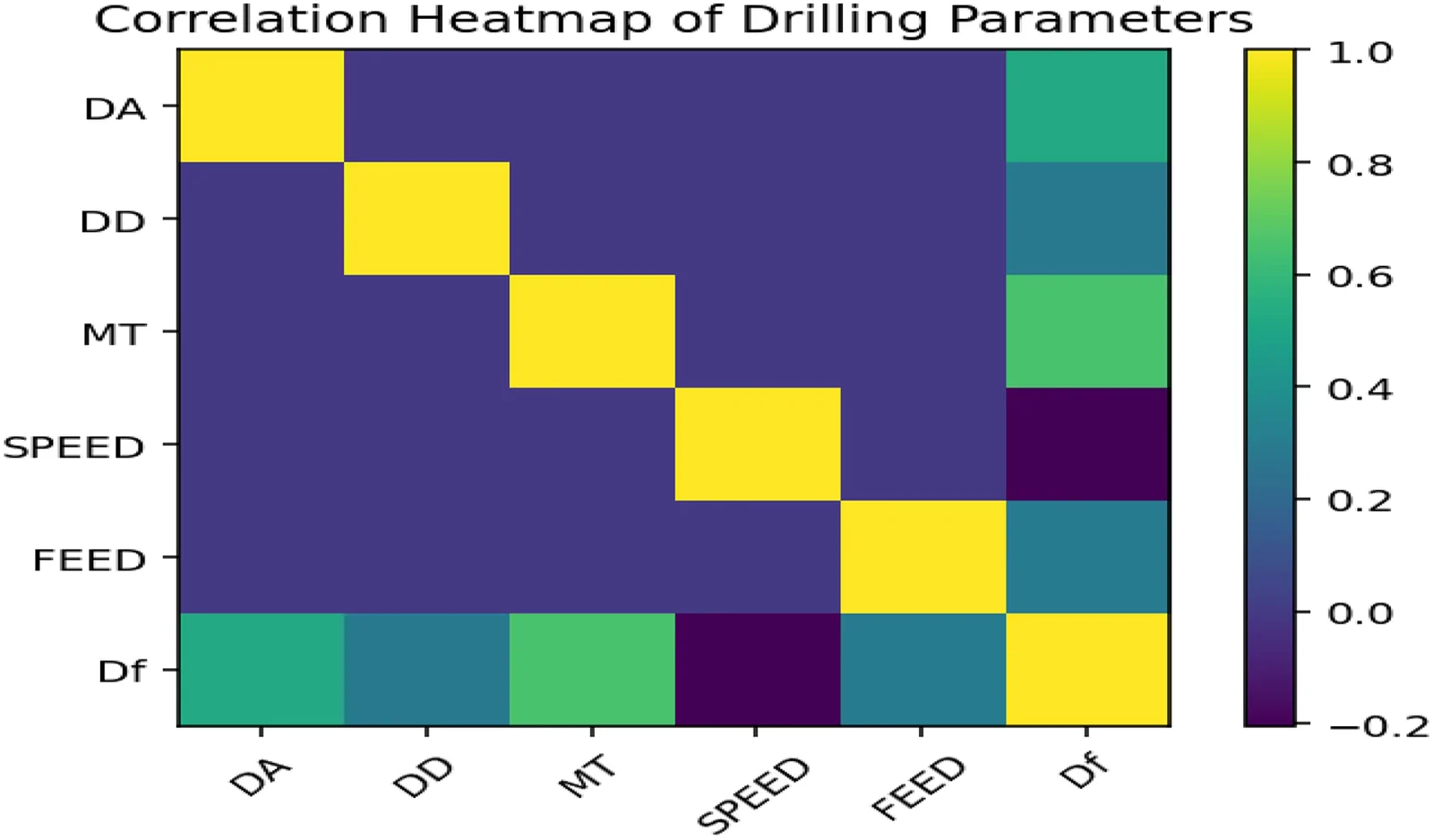
Correlation heatmap.

The Pearson correlation coefficients for the drilling parameters are illustrated in the heatmap shown in Fig 10. This analysis reveals that Material Thickness (MT) and Drill Angle (DA) have the most significant positive influence on the response variable, Df, as indicated by the light green areas in the correlation matrix. Conversely, Spindle Speed (SPEED) exhibits a noticeable negative correlation with Df, suggesting an inverse relationship where higher rotational speeds result in a decrease in the measured output. Additionally, the parameters Drill Diameter (DD) and Feed Rate (FEED) show relatively low correlation values, indicating they have a minor impact within the established experimental conditions. Importantly, the near-zero correlation observed between the independent variables (represented by dark blue cells) confirms that there is no multicollinearity in the dataset. This independence ensures that subsequent statistical modeling and optimization can accurately attribute changes in Df to variations in individual parameters without any confounding effects.

**Fig 11:**
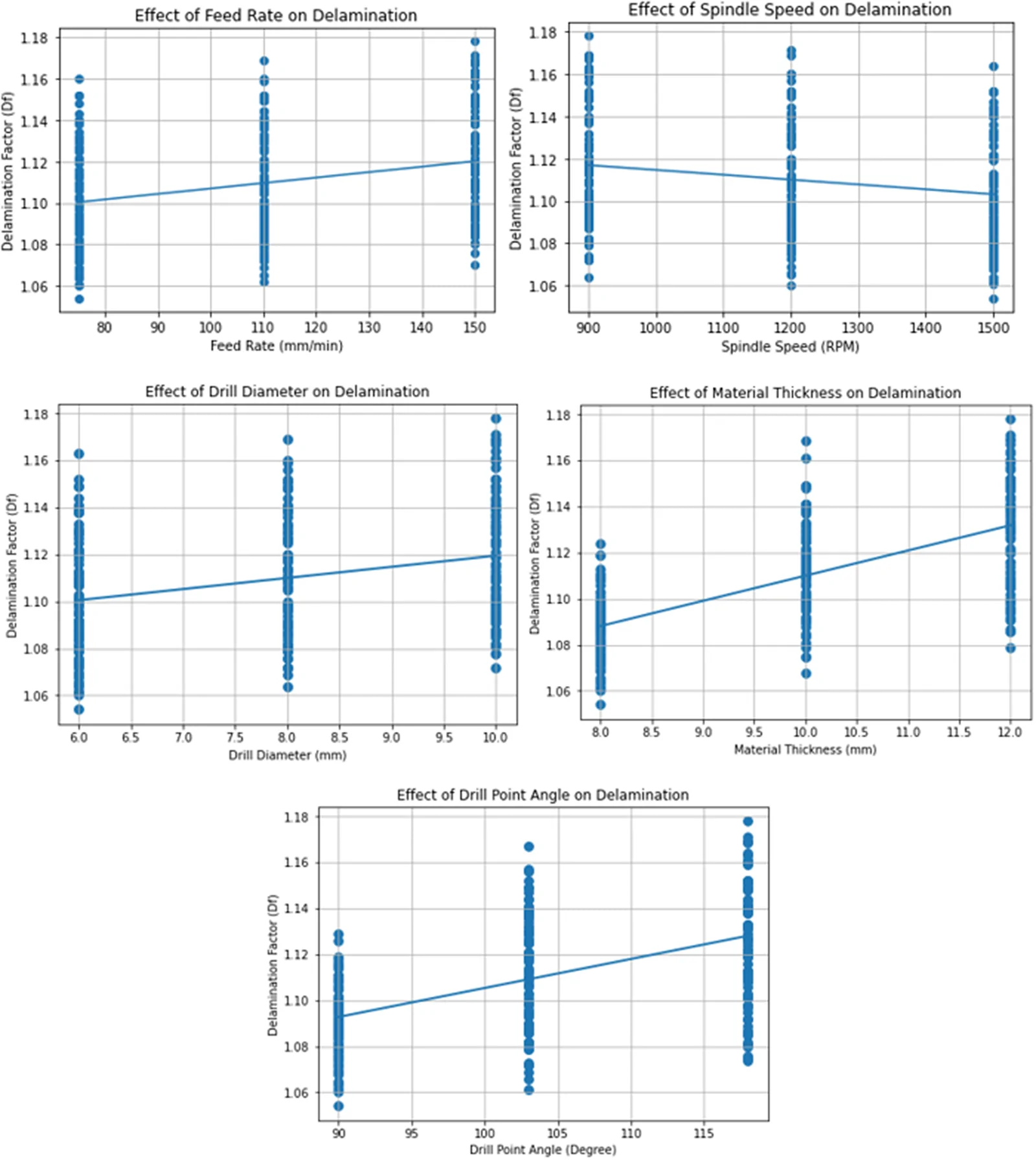
Main effect plots of drilling parameters on delamination factor.

The influence of individual drilling parameters on the delamination factor is illustrated in Fig 11, which presents the main effect plots for feed rate, drill diameter, material thickness, spindle speed, and drill point angle. These plots provide a clear understanding of the trend and relative significance of each parameter.

From Fig 11, it is evident that feed rate has the most pronounced effect on delamination. As the feed rate increases from 75 to 150 mm/min, the delamination factor shows a consistent upward trend. This behavior is attributed to the increase in thrust force generated at higher feed rates, which promotes interlaminar crack propagation and results in greater damage.

The effect of drill diameter also shows a positive correlation with delamination. Larger drill diameters (10 mm) produce higher delamination compared to smaller diameters (6 mm). This is due to the increased cutting area, which leads to higher thrust forces and consequently more severe interlaminar stresses.

Similarly, material thickness significantly influences delamination. As the thickness increases from 8 mm to 12 mm, the delamination factor increases noticeably. Thicker laminates require higher penetration force, which elevates thrust force and enhances the likelihood of delamination, particularly at the exit side of the hole.

In contrast, spindle speed exhibits a negative relationship with delamination. Increasing the spindle speed from 900 rpm to 1500 rpm results in a slight reduction in delamination factor. This can be attributed to improved cutting efficiency and reduced cutting resistance at higher speeds, which lowers the thrust force and minimizes damage.

The drill point angle also affects delamination, with higher angles (118°) leading to increased damage compared to lower angles (90°). This is because larger point angles increase the chisel edge length, thereby generating higher thrust forces during drilling.

Delamination during the drilling of composite laminates is significantly influenced by machining parameters, with the feed rate being the most critical factor. When the feed rate is increased, it results in a higher thrust force that can exceed the interlaminar bond strength, promoting crack propagation between the layers and leading to severe delamination [31]. The drill diameter also plays an important role; a larger diameter increases the cutting area and, consequently, the thrust force, which exacerbates damage at the hole exit [32]. Additionally, the thickness of the laminate contributes to delamination, as thicker composites require greater penetration force, resulting in higher stresses across the plies [33].

The thrust force produced during machining, which is governed by a number of process variables, is the main factor influencing drilling-induced delamination in composite laminates. The most important factor influencing delamination behavior among these is the feed rate. Higher thrust forces result from an increase in the amount of material removed every revolution as the feed rate rises. This force starts and spreads cracks across the layers when it exceeds the composite’s interlaminar fracture toughness, greatly raising the delamination factor [32,33].

Spindle speed is inversely related to delamination damage. Higher cutting speeds enhance the shearing action at the tool-workpiece interface and can cause localized thermal softening of the matrix material. This softening reduces cutting resistance and, in turn, lowers the thrust force, thereby minimizing delamination. Therefore, operating at higher spindle speeds is generally advantageous for improving the quality of holes in composite drilling.

Drill diameter is a crucial factor that affects delamination. When the drill diameter increases, the area of contact between the cutting tool and the workpiece also expands. This results in higher cutting and thrust forces. The increase in force heightens interlaminar stresses, making the laminate more vulnerable to damage, especially in the exit region of the hole [33,34]. Consequently, larger drill diameters are generally associated with higher delamination factors compared to smaller diameters.

The drill point angle further affects the damage mechanism; a higher point angle increases the chisel edge length, producing greater thrust force and thus more pronounced delamination [35]. In contrast, spindle speed has an inverse relationship with delamination, where higher cutting speeds reduce thrust force due to thermal softening and improved cutting action, thereby minimizing interlaminar failure [36]. These observations are consistent with established findings in composite machining literature, where thrust force is identified as the primary driver of delamination damage.

Among the investigated parameters, feed rate, material thickness, and drill point angle all exhibit a positive influence on the delamination factor. The Pearson correlation analysis indicates that material thickness shows the strongest overall linear correlation with delamination, while the main-effects analysis demonstrates that increasing feed rate produces the largest practical increase in the average delamination factor within the investigated operating range. Therefore, feed rate is identified as the most influential controllable machining parameter from a process optimisation perspective.

**Fig 12:**
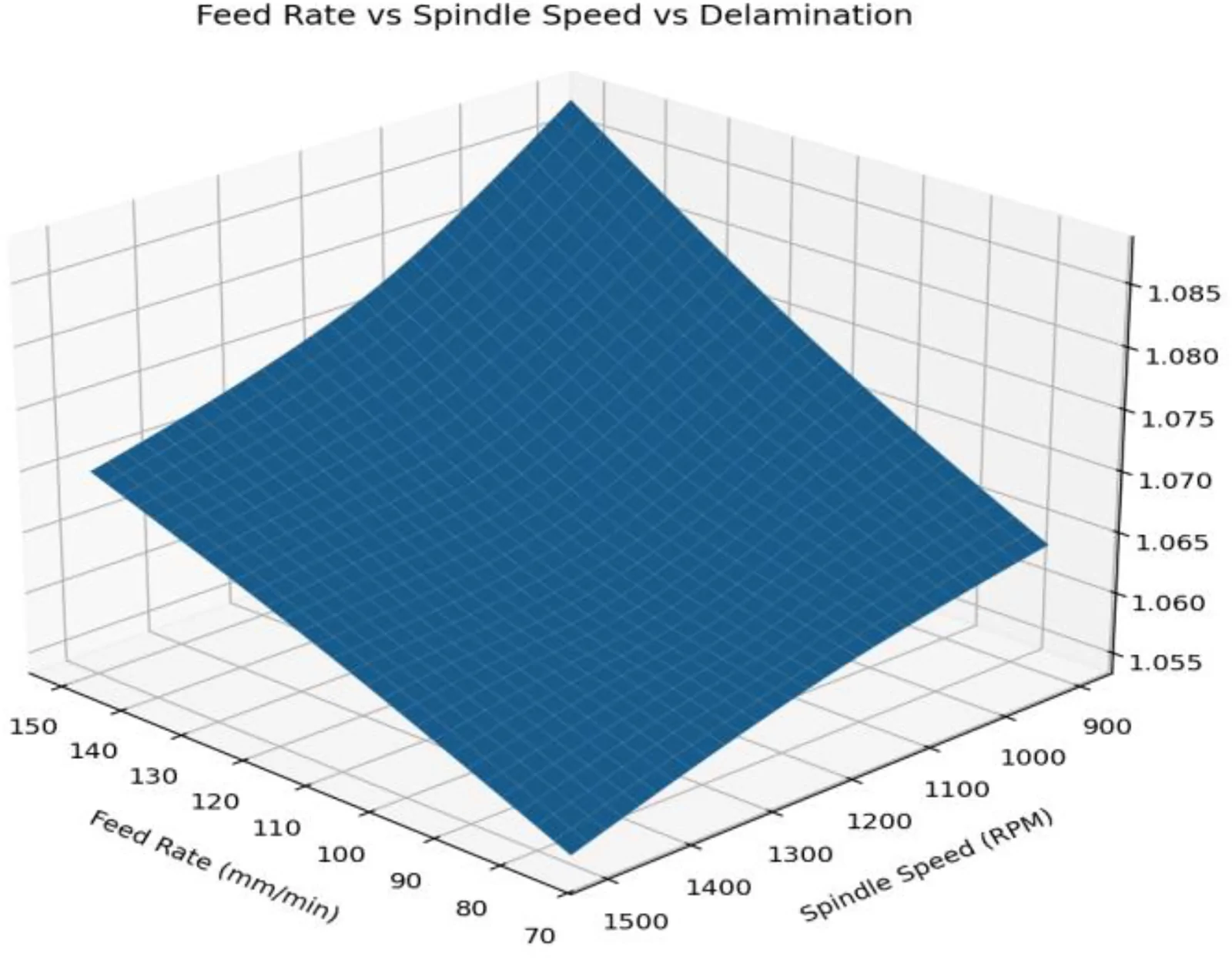
Interaction effect of feed rate and spindle speed on delamination factor.

The combined influence of feed rate and spindle speed on the delamination factor is illustrated in Fig 12. The 3D surface plot clearly shows the interaction between these two critical drilling parameters and their effect on damage formation. The Fig illusrates that the delamination factor significantly increases with a higher feed rate, while it decreases with an increase in spindle speed. At lower feed rates (approximately 75 mm/min) and higher spindle speeds (1500 rpm), the delamination factor reaches its minimum value. This combination of conditions promotes a smoother cutting action and reduces thrust force, thereby minimizing interlaminar damage. Conversely, at higher feed rates (150 mm/min) and lower spindle speeds (900 rpm), the delamination factor reaches its maximum. This is due to the combined effect of increased material removal rate and reduced cutting efficiency, which results in higher thrust forces and severe delamination.

The surface trend demonstrates a nearly linear increase in delamination along the feed rate axis and a decreasing trend along the spindle speed axis. This confirms that feed rate has a dominant effect, while spindle speed acts as a controlling parameter that can mitigate the damage.

**Fig 13:**
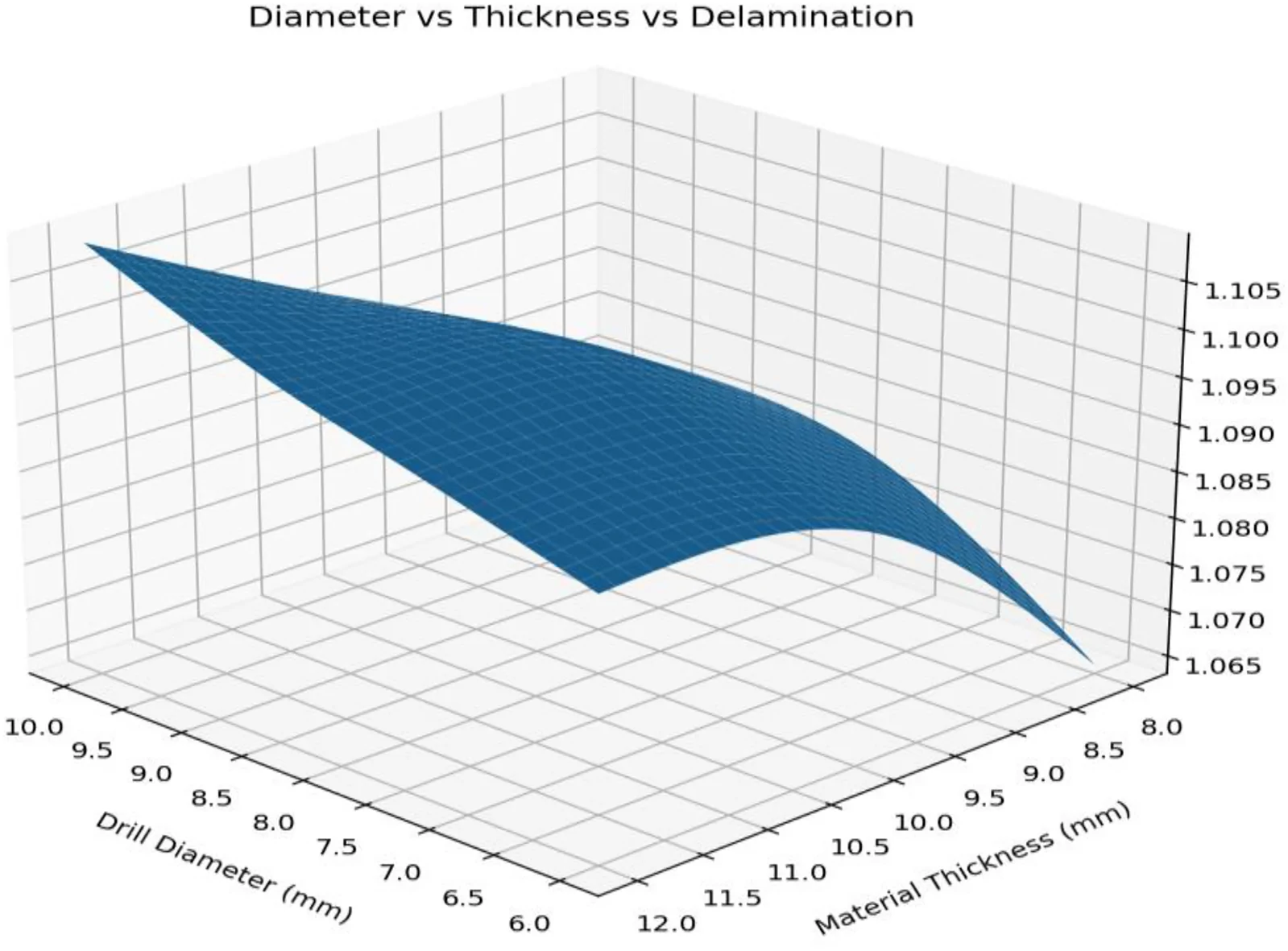
Interaction effect of drill diameter and material thickness on delamination factor.

The interaction effect of drill diameter and material thickness on the delamination factor is presented in Fig 13. The 3D surface plot illustrates how the combined variation of these two parameters influences the extent of drilling-induced damage in GFRP composites.

Fig 13 clearly shows that the delamination factor increases with both drill diameter and material thickness. At lower values, such as a diameter of 6 mm and a thickness of 8 mm, the delamination factor is minimal. This is attributed to the reduced cutting area and the lower resistance provided by thinner laminates, which leads to a decrease in thrust force generation.

As the diameter of the drill increases, the contact area between the tool and the workpiece also grows, resulting in higher cutting forces. Likewise, increasing the thickness of the material necessitates greater penetration force, which further raises the thrust force. The combined effect of these two factors significantly increases interlaminar stresses, thereby promoting the risk of delamination.

The surface plot reveals a smooth and gradual increase in delamination along both axes, indicating a significant positive interaction between drill diameter and thickness. The highest delamination values occur at the maximum combination of diameter (10 mm) and thickness (12 mm), which correlates with the expected peak thrust force.The surface plot reveals a smooth and gradual increase in delamination along both axes, indicating a significant positive interaction between drill diameter and thickness. The highest delamination values occur at the maximum combination of diameter (10 mm) and thickness (12 mm), which correlates with the expected peak thrust force.

The implementation of Physics-Informed Neural Networks (PINNs) offers a robust and reliable approach for predicting delamination behavior. The created PINN model yields high prediction accuracy, typically with an R2 value greater than 0.98, by combining experimental data with governing physical rules. This shows that the experimental and projected findings agree very well. The model is an effective tool for process optimization and damage minimization in composite drilling applications because it captures the intricate nonlinear interactions between machining parameters and delamination [37,38].

### 3.6 Ablation study of Physics-Informed Neural Network (PINN)

An ablation study was conducted by comparing the proposed Physics-Informed Neural Network (PINN) with a conventional data-driven Artificial Neural Network (ANN) of identical network structure but without the physics-based loss component to assess the impact of physics-based constraints on the model’s predictive capability. The main goal was to quantify how embedded physical laws affect prediction accuracy, convergence stability, and generalization performance. In this study, the PINN incorporates the governing thrust-force-induced delamination mechanics into training via an additional physics loss term, whereas the conventional ANN minimizes only the data-driven prediction error. This comparison clearly demonstrates the effectiveness of physics-guided learning for predicting drilling-induced delamination. For this study, two separate models were developed and trained using the same experimental dataset consisting of 243 drilling trials.

The first model was a purely data-driven Artificial Neural Network (ANN) trained using only experimental input–output relationships. For ANN model, Drill point angle, Drill diameter, Material thickness, Spindle speed and Feed rate are selcted as input parameters. The chosen output parameters is delamination factor. The network architecure consists of input layer of 5 neorons, hidden layer of 64-64-32 neorons and output layer of 1neoron.

The Loss function: $L_{ANN=}\;L_{data}$

Where:


$$\begin{array}{c} L_{data}=\frac{\text{1}}{N}\sum_{i=\text{1}}^{N}({Df,pred-Df,exp)}^{\text{2}} \end{array}$$

The second model was the proposed Physics-Informed Neural Network (PINN), which combines experimental data with the governing equations of drilling mechanics. The architecture and dataset used in this method are similar to those of the ANN, but an additional physics-based constraint is incorporated into the loss function.

The total loss function of the PINN is defined as:


$$\begin{array}{c} L=L_{Df}+\lambda_{\text{1}}L_{Ft}+\lambda_{\text{2}}L_{physics}, \end{array}$$

Where;


$$\begin{array}{c} L_{physics}=\frac{\text{1}}{N}\sum_{i=\text{1}}^{N}{\left({\hat{D}}_{f,i}-D_{f,i}^{phys}\right)}^{\text{2}} \end{array}$$

The physics loss term ensures that the predicted delamination behaviour remains consistent with the governing thrust-force-induced fracture mechanics.

The ANN and PINN predicted delamination for each experiment is presented in Table 3. The predictive performance of both models was evaluated using the coefficient of determination (R²), and Root Mean Square Error (RMSE) and presented in Table 4. The results clearly indicate that the incorporation of physical constraints significantly improved the predictive capability of the model. The PINN achieved a higher R² value and substantially lower RMSE values compared with the conventional ANN model.

**Table 3:** Experimental, ANN predicted and PINN predicted values for each trial.

Exp. No.	DA	DD	MT	SPEED	FEED	Df(Exp)	ANN	PINN
1	90	10	10	900	75	1.095	1.0877	1.0981
	.	.	.	.	.	.	.	.
102	103	6	10	1200	150	1.118	1.1148	1.1190
.	.	.	.	.	.	.	.	.
243	118	10	12	1500	75	1.103	1.1137	1.1099

**Table 4:** Comparative performance of ANN and PINN models.

Model	R^2^	RMSE
ANN (without physics constraints)	0.9422	0.0075
PINN (with physics constraints)	0.9769	0.0048

#### Discussion on ablation results.

The ablation study shows that physics-guided learning plays a significant role in improving drilling-induced delamination prediction. While the conventional ANN model could capture the nonlinear relationship between drilling parameters and the delamination factor, its predictions relied solely on patterns in the experimental data and lacked physical consistency. In contrast, the proposed PINN model directly integrated the governing mechanics of thrust-force-induced delamination into the learning process. This added physics-based regularization constrained the solution space, preventing physically unrealistic predictions and thereby enhancing the model’s robustness and generalization ability.

### Scientific significance of ablation study

The ablation analysis confirms that the proposed model’s superior performance stems primarily from integrating physics-based constraints into the learning framework, rather than just neural network complexity. The results demonstrate that:

- Physics-informed learning enhances predictive reliability.
- Governing mechanics improve model interpretability.
- PINNs reduce reliance on large experimental datasets.
- Physics-based constraints boost generalization capability.
- The proposed framework overcomes key limitations of conventional black-box machine learning methods.

Therefore, the proposed PINN framework offers a robust and physically interpretable solution for intelligent prediction of drilling-induced delamination in composite materials.

### Comparative performance of ANN and PINN models

**Fig 14:**
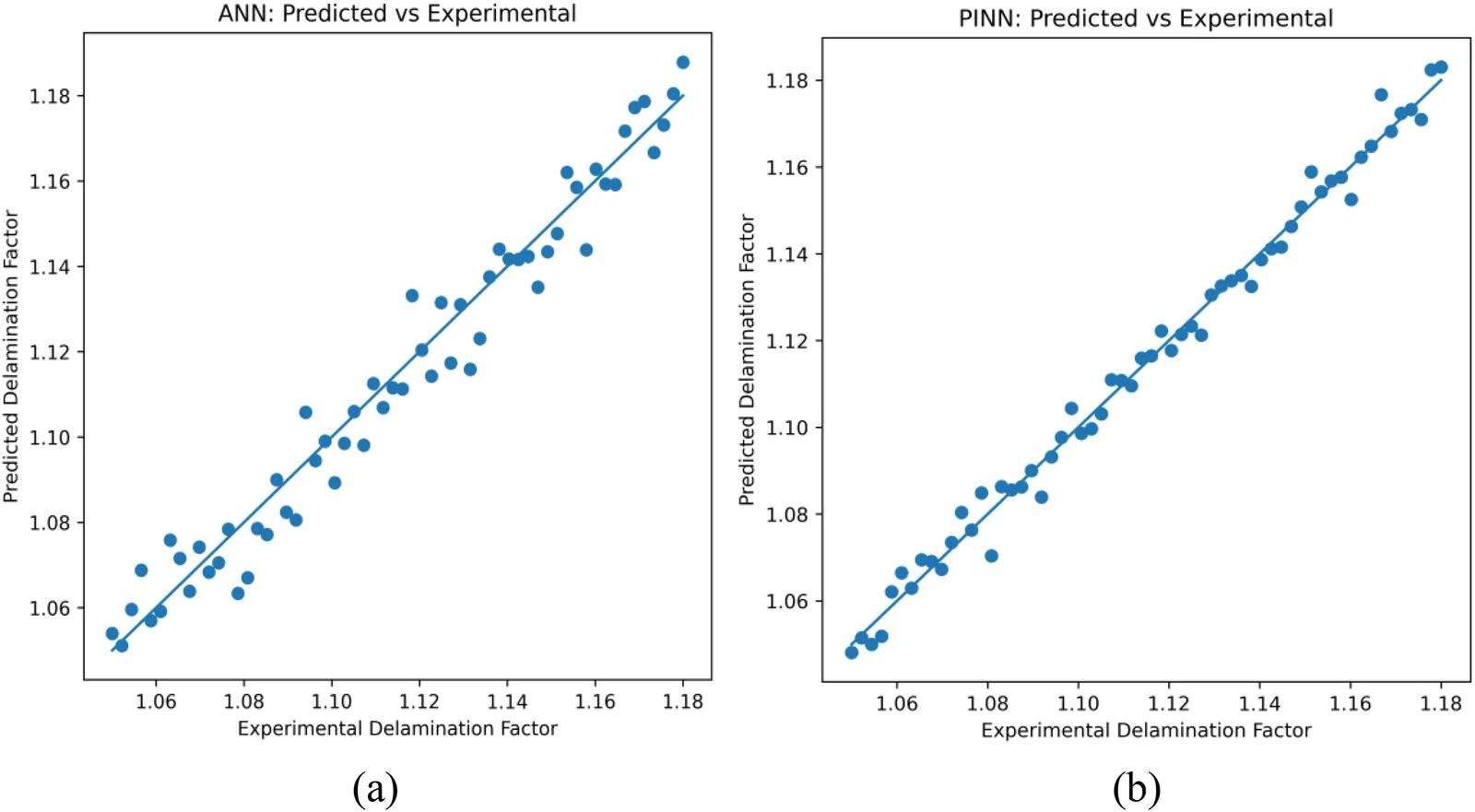
ANN Predicted vs Experimental results (a) and PINN Predicted vs experiemtnal results (b).

The ANN model shows reasonable agreement between predicted and experimental delamination values. However, the noticeable scatter away from the 45° reference line indicates larger prediction deviations and diminished generalization capability. The obtained results are represented in Fig 14 (a).

The PINN model demonstrates stronger agreement between predicted and experimental values, with most data points closely aligned along the 45° reference line. This confirms the enhanced prediction accuracy achieved through physics-guided learning. The results ottained are illustrated in Fig 14 (b).

## Conclusions

This study developed a predictive framework based on Physics-Informed Neural Networks (PINNs) to evaluate drilling-induced delamination in Glass Fiber Reinforced Polymer (GFRP) composites. A comprehensive full factorial experimental design comprising 243 drilling trials was conducted to investigate the effects of drill point angle, drill diameter, laminate thickness, spindle speed, and feed rate on delamination behavior.

Among the investigated parameters, feed rate, material thickness, and drill point angle all exhibit a positive influence on the delamination factor. The Pearson correlation analysis indicates that material thickness shows the strongest overall linear correlation with delamination, while the main-effects analysis demonstrates that increasing feed rate produces the largest practical increase in the average delamination factor within the investigated operating range. Therefore, feed rate is identified as the most influential controllable machining parameter from a process optimisation perspective.

The developed PINN model effectively captured the complex nonlinear relationship between drilling parameters and delamination behavior while maintaining the physical consistency of the process. By incorporating thrust-force-induced delamination mechanics into the loss function, the proposed framework achieved a high prediction accuracy with an R² value of 0.9769 and a low root mean square error (RMSE) of 0.0048. The convergence behavior, residual distribution, and sensitivity analyses verified the robustness, stability, and good interpolation for the considered range of the developed model.

In comparison to conventional purely data-driven approaches, the proposed physics-guided learning framework offers improved interpretability, enhanced prediction reliability, and a reduced dependence on large experimental datasets. The integration of physical constraints delaienables the model to remain consistent with the underlying mechanics of composite damage evolution, thus addressing one of the major limitations of traditional black-box machine learning techniques.

The findings of this study demonstrate the feasibility of employing PINNs for intelligent machining applications involving composite materials. The proposed methodology serves as an effective tool for predictive modeling, process optimization, and damage minimization in advanced composite drilling operations. Additionally, the framework can be extended to other machining processes, composite systems, and hybrid manufacturing environments to develop next-generation physics-guided smart manufacturing solutions.

The ablation study confirmed that incorporating thrust-force-based physical constraints significantly improved the predictive capability of the proposed PINN model. Compared to the conventional ANN framework, the PINN delivered higher prediction accuracy, reduced error metrics, enhanced convergence stability, and better generalization performance. These results highlight the effectiveness of physics-guided learning for intelligent machining applications involving composite materials.

However, this study is limited to GFRP laminates under dry drilling conditions and does not consider tool wear progression, thermal effects, or real-time monitoring signals. Future work will focus on integrating multi-physics effects, tool condition monitoring, transfer learning strategies, and hybrid FEM–PINN frameworks to further enhance predictive capability and industrial applicability in intelligent composite manufacturing.
